# Supplementary material for: Prenatal and postnatal environmental influences on child growth trajectories across a rural-urban gradient: an analysis of the ECoMiD longitudinal birth cohort
Source: medRxiv. 2025 Nov 13:2025.11.10.25339957. Preprint. [Version 1] doi: 10.1101/2025.11.10.25339957 (PMC12642747; doi:10.1101/2025.11.10.25339957)
Supplement: Supplement 1 [file media-1.pdf]

Supplementary Information

Supplementary methods

Contamination variables

Handrinses were performed on mothers and children in 100 mL of distilled water in a 69-oz Whirlpak bag. Samples of drinking water (100 mL) were taken from the source and the same assays were performed as on handrinses after sodium thiosulfate added to neutralize any chlorine. For each survey timepoint, samples were collected on three non-consecutive days (all within one week of each other) and were measured for *E.coli* using Colilert and Petrifilm. Samples were categorized into 1-5 ordinal score for *E.coli* levels depending on Colilert and Petrifilm results (see table below). The score for all three measurements was averaged and re-binned into a semi-continuous score that approximates the log scale of concentration of *E.coli*. Semi-continuous scores were then averaged across timepoints within households.

**Colilert result**	**Petrifilm result**	**Concentration of E.coli per 100ml**	**Ordinal score (assigned for each sample [3 per timepoint])**	**Semi-continuous score (assigned to average of 3 samples per timepoint)**
Absent	0	<1 CFU or MPN/100 ml	1	1
Present	0	1-99 MPN/100 ml	2	2.5
Present	1-9 CFU/1 ml	100-999 CFU/100 ml	3	4
Present	10-49 CFU/1 ml	1000-4999 CFU/100ml	4	5
Present	≥50 CFU/1ml	≥5000 CFU/100ml	5	6

Figure S1. Exposure variable collection timepoints and imputation strategy.

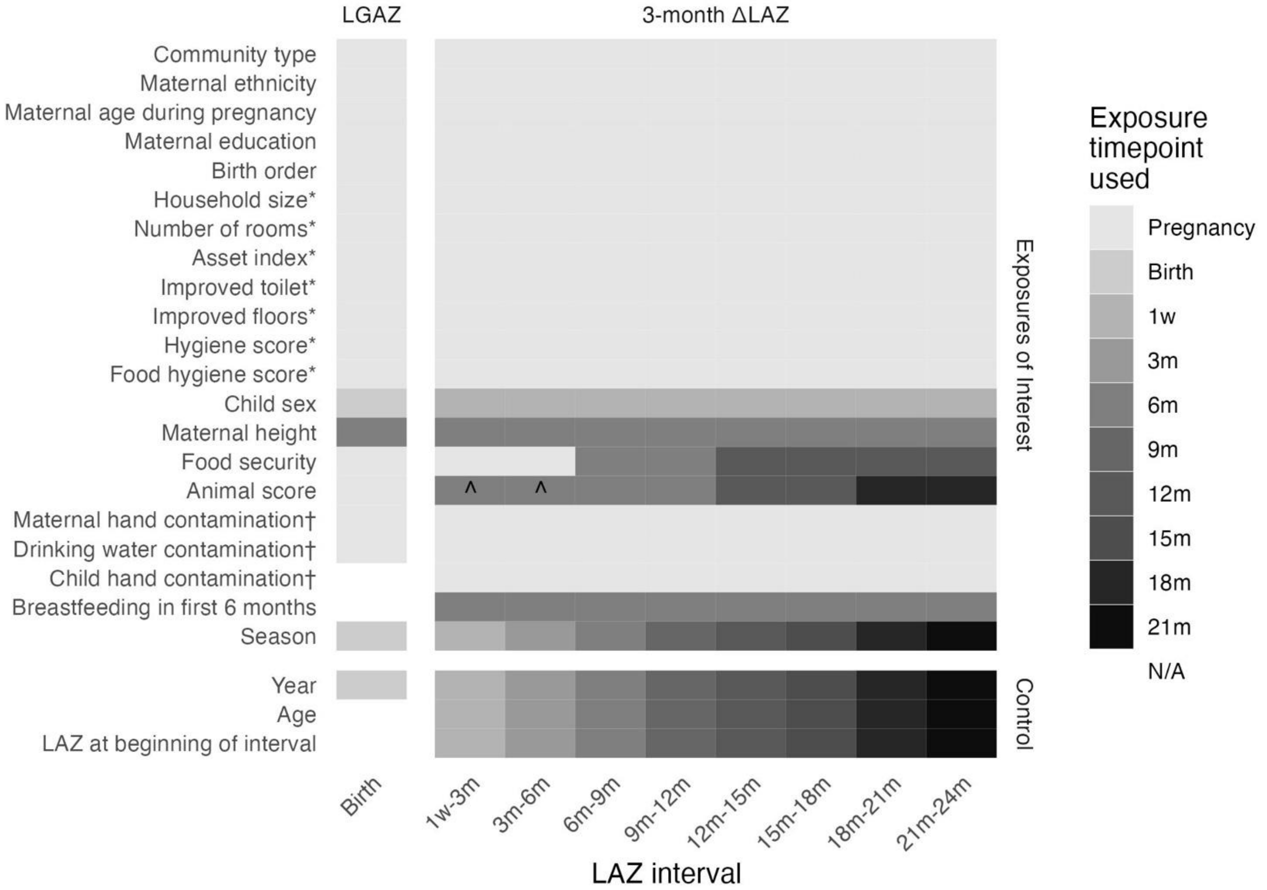

Notes: *Recollected if household moved but utilized pregnancy data only; ^Maternal exposure data; †Averaged over all visits

Table S1. Descriptive statistics of time-invariant exposure and outcome variables.

	Overall<br>n = 401	Urban<br>n = 102	Intermediate<br>n = 115	Rural – road<br>n = 142	Rural – river<br>n = 42
Enabling Determinants – Norms					
Child is male	201 (51%)	54 (54%)	57 (50%)	65 (46%)	25 (60%)
Missing	5	2	2	1	0
Birth order					
1	108 (27%)	29 (28%)	38 (33%)	32 (23%)	9 (21%)
2	121 (30%)	37 (36%)	38 (33%)	35 (25%)	11 (26%)
3+	172 (43%)	36 (35%)	39 (34%)	75 (53%)	22 (52%)
Maternal age					
Less than 22 yrs	87 (22%)	22 (22%)	35 (30%)	23 (16%)	7 (17%)
22-29 years	210 (52%)	52 (51%)	55 (48%)	85 (60%)	18 (43%)
30 years and more	104 (26%)	28 (27%)	25 (22%)	34 (24%)	17 (40%)
Mom identifies as Afro-Ecuadorian	290 (72%)	51 (50%)	72 (63%)	126 (89%)	41 (98%)
Enabling Determinants – Resources					
Household wealth quartile					
Q1	104 (26%)	21 (21%)	39 (34%)	34 (24%)	10 (24%)
Q2	97 (24%)	22 (22%)	26 (23%)	34 (24%)	15 (36%)
Q3	96 (24%)	21 (21%)	17 (15%)	47 (33%)	11 (26%)
Q4	104 (26%)	38 (37%)	33 (29%)	27 (19%)	6 (14%)
Maternal height (cm)	160 (155, 164)	160 (156, 164)	158 (154, 164)	160 (155, 166)	158 (153, 162)
Missing	37	18	10	8	1
Maternal education					
Primary or less	40 (10.0%)	2 (2.0%)	10 (8.7%)	17 (12%)	11 (26%)
Lower secondary	60 (15%)	3 (2.9%)	15 (13%)	27 (19%)	15 (36%)
Upper secondary	220 (55%)	79 (77%)	57 (50%)	71 (50%)	13 (31%)
Post-secondary or greater	81 (20%)	18 (18%)	33 (29%)	27 (19%)	3 (7.1%)
Underlying Determinants – Services					
Number of rooms in household	3 (2, 3)	3 (2, 3)	3 (2, 4)	2 (2, 3)	3 (2, 4.75)
Household size	4 (3, 6)	5 (3, 6)	4 (3, 6)	4 (3, 5)	4 (3, 6)
Household has improved floor	270 (67%)	90 (88%)	58 (50%)	111 (78%)	11 (26%)
Household has improved toilet	375 (95%)	101 (100%)	108 (96%)	137 (98%)	29 (69%)
Missing	5	1	2	2	0
Underlying Determinants – Practices					
Hygiene score	7 (5, 8)	8 (5, 8)	6 (5, 8)	7 (5, 8)	8 (8, 8)
Missing	5	3	2	0	0
Food covering score	3 (3, 3)	3 (3, 3)	3 (2, 3)	3 (3, 3)	3 (3, 3)
Missing	19	18	1	0	0
Average maternal hand contamination	2.0 (2.0, 2.5)	2.5 (2.0, 2.5)	2.5 (2.0, 2.5)	2.0 (2.0, 2.5)	1.5 (1.0, 1.5)
Missing	46	8	9	21	8
Underlying Determinants – Food					
Household is moderately/severely food insecure during pregnancy	144 (36%)	22 (22%)	33 (29%)	81 (57%)	8 (19%)
Missing	1	0	0	1	0
Child was exclusively breastfed for >50% of days 0-6m	211 (54%)	58 (58%)	54 (48%)	65 (49%)	34 (81%)
Missing	13	2	2	9	0
Average drinking water contamination	2.5 (2.0, 2.5)	2.5 (2.5, 2.5)	2.5 (2.0, 2.5)	2.0 (2.0, 2.5)	1.5 (1.5, 2.0)
Missing	46	8	9	21	8
Immediate Determinants – Care					
Average child hand contamination	2.5 (1.75, 2.5)	2.5 (2.5, 2.5)	2.5 (2.5, 2.5)	2.5 (1.75, 2.5)	1.0 (1.0, 1.0)
Missing	77	24	16	28	9
Outcomes					
Length-for-gestational-age Z-score, mean (SD)	0.73 (1.27)	0.61 (1.30)	0.54 (1.27)	0.92 (1.16)	0.94 (1.45)
Missing	22	3	9	2	8
Average 3-month ΔLAZ, mean (SD)	-0.13 (0.81)	-0.09 (0.76)	-0.13 (0.79)	-0.13 (0.82)	-0.24 (-0.89)

Note: Summary statistics presented are n (%) and median (IQR) unless noted otherwise

Table S2. Descriptive statistics of time-varying exposure and outcome variables (n = 401)

	**Pregnancy**	**1w**	**3m**	**6m**	**9m**	**12m**	**15m**	**18m**	**21m**	**24m**
Household is moderately/severely food insecure	144 (36%)	144 (36%)	144 (36%)	129 (32%)	129 (32%)	133 (38%)	133 (38%)	132 (37%)	132 (37%)	126 (39%)
Missing	1	1	1	0	0	47	47	47	47	81
Animal exposure score	1.61 (0.00, 3.51)	0.48 (0.00, 2.29)	0.48 (0.00, 2.29)	0.48 (0.00, 2.29)	0.48 (0.00, 2.29)	1.35 (0.39, 3.02)	1.35 (0.39, 3.02)	1.67 (0.75, 2.75)	1.67 (0.75, 2.75)	1.58 (0.71, 2.45)
Missing	0	34	34	34	34	46	46	70	70	89
LAZ	-	0.33 (-0.45, 1.04)	0.21 (-0.62, 1.00)	0.01 (-0.80, 0.75)	-0.36 (-1.10, 0.49)	-0.42 (-1.34, 0.40)	-0.67 (-1.60, 0.05)	-0.85 (-1.68, -0.07)	-1.06 (-1.73, -0.16)	-0.80 (-1.47, 0.03)
Missing	401	12	42	55	65	69	75	100	96	98

Note: Summary statistics presented are n (%) and median (IQR) unless noted otherwise

Table S3. Unadjusted associations between community, demographic, and environmental exposures with child anthropometry at birth and child postnatal growth trajectories.

	LGAZ			3-month ΔLAZ		
	n	MD	95% CI	n	MD	95% CI
Enabling Determinants – Governance						
Community type	378			2,477		
Urban		—	—		—	—
Intermediate		-0.01	-0.34, 0.33		-0.03	-0.08, 0.02
Rural – road		0.31	0.00, 0.62		-0.04	-0.09, 0.01
Rural – river		0.34	-0.13, 0.81		-0.15	-0.26, -0.05
Enabling Determinants – Norms						
Child is male	378	-0.11	-0.35, 0.14	2,477	0.01	-0.03, 0.05
Birth order	378			2,477		
1		—	—		—	—
2		-0.23	-0.55, 0.10		0.05	0.00, 0.10
3+		-0.01	-0.31, 0.30		0.01	-0.04, 0.06
Maternal age during pregnancy	378			2,477		
30 years and more		—	—		—	—
Less than 22 yrs		-0.04	-0.40, 0.31		0.01	-0.04, 0.07
22-29 years		0.07	-0.22, 0.37		0.00	-0.05, 0.05
Mom identifies as Afro-Ecuadorian	378	0.29	0.02, 0.56	2,477	-0.01	-0.05, 0.04
Enabling Determinants – Resources						
Household wealth quartile	378			2,477		
Q1		—	—		—	—
Q2		0.03	-0.32, 0.38		0.03	-0.03, 0.09
Q3		-0.10	-0.45, 0.24		0.03	-0.03, 0.09
Q4		-0.13	-0.47, 0.21		0.08	0.03, 0.13
Maternal height (per 5 cm)	349	0.15	0.06, 0.24	2,458	0.01	0.00, 0.03
Maternal education	378			2,477		
Primary or less		—	—		—	—
Lower secondary		-0.04	-0.56, 0.47		-0.09	-0.18, 0.00
Upper secondary		-0.01	-0.44, 0.42		-0.04	-0.11, 0.03
Post-secondary or greater		0.01	-0.48, 0.49		-0.05	-0.12, 0.02
Season	378			2,477		
Late dry		—	—		—	—
Early rainy		0.15	-0.25, 0.54		0.04	-0.06, 0.14
Late rainy		-0.10	-0.46, 0.25		0.07	-0.03, 0.16
Early dry		-0.11	-0.46, 0.23		0.00	-0.10, 0.09
Underlying Determinants – Services						
Number of rooms in household	378	0.00	-0.09, 0.09	2,477	-0.01	-0.03, 0.01
Household size	378	0.00	-0.05, 0.05	2,477	0.00	-0.01, 0.01
Household has improved floor	378	0.18	-0.09, 0.44	2,477	0.01	-0.04, 0.05
Household has improved toilet	374	0.28	-0.31, 0.87	2,462	-0.04	-0.16, 0.08
Underlying Determinants – Practices						
Hygiene score	374	-0.01	-0.08, 0.05	2,474	0.00	-0.01, 0.01
Food hygiene score	361	-0.07	-0.23, 0.09	2,410	0.01	-0.02, 0.03
Animal exposure score	378	-0.04	-0.16, 0.07	2,473	-0.02	-0.04, 0.00
Average maternal hand contamination	333	0.01	-0.28, 0.29	2,194	0.05	-0.01, 0.11
Underlying Determinants – Food						
Moderately/severely food insecure	377	-0.03	-0.29, 0.23	2,473	0.05	0.00, 0.09
Child was exclusively breastfed for >50% of days 0-6m	370	0.02	-0.23, 0.27	2,453	0.00	-0.04, 0.04
Average drinking water contamination	333	-0.23	-0.52, 0.05	2,194	0.02	-0.03, 0.07
Immediate Determinants – Care						
Average child hand contamination				2,178	0.07	0.01, 0.12

Note: MD = mean difference; CI = confidence interval; LGAZ = length-for-gestational-age Z-score; ΔLAZ = change in length-for-age Z-score.

Table S4. Effect modification of the association between community, demographic, and environmental exposures and birth anthropometry by community type.

			Stratified Estimates			
Variable	Contrast	Global p-value	Urban	Intermediate	Rural - road	Rural - river
Enabling Determinants – Norms						
Child is male		0.22	-0.25 (-0.78, 0.28)	-0.57* (-1.06, -0.09)	0.01 (-0.4, 0.43)	0.24 (-0.62, 1.1)
Birth order	2	0.12	-0.41 (-1.08, 0.26)	0.14 (-0.46, 0.74)	-0.57 (-1.2, 0.06)	0.11 (-1.02, 1.24)
	3+		-0.32 (-1.04, 0.4)	0.27 (-0.38, 0.93)	-0.33 (-0.91, 0.26)	1.18* (0.1, 2.26)
Maternal age during pregnancy	22-29 years	0.11	0.56 (-0.06, 1.19)	-0.1 (-0.72, 0.52)	0.31 (-0.2, 0.83)	-0.68 (-1.63, 0.27)
	Less than 22 yrs		0.33 (-0.45, 1.11)	0.07 (-0.66, 0.8)	0.34 (-0.38, 1.07)	-1.4* (-2.59, -0.22)
Mom identifies as Afro-Ecuadorian		0.21	-0.12 (-0.65, 0.41)	-0.16 (-0.66, 0.35)	0.69* (0.02, 1.36)	0.27 (-2.17, 2.71)
Enabling Determinants – Resources						
Household wealth quartile	Q2	0.69	-0.23 (-1.05, 0.58)	0.11 (-0.56, 0.79)	0.33 (-0.29, 0.94)	-0.03 (-1.09, 1.03)
	Q3		-0.24 (-1.05, 0.57)	0.23 (-0.54, 1)	-0.21 (-0.78, 0.37)	-0.15 (-1.38, 1.07)
	Q4		-0.41 (-1.13, 0.31)	-0.34 (-0.96, 0.27)	0.32 (-0.34, 0.98)	-0.44 (-1.78, 0.9)
Maternal height (per 5 cm)		0.28	0.03 (-0.22, 0.28)	0.16 (-0.03, 0.35)	0.18* (0.04, 0.33)	0.39** (0.12, 0.66)
Maternal education	Lower secondary	0.14	2.4* (0.05, 4.76)	-0.3 (-1.36, 0.76)	0.21 (-0.56, 0.98)	-0.18 (-1.36, 1)
	Upper secondary		1.83* (0.12, 3.54)	-0.37 (-1.25, 0.51)	0.53 (-0.16, 1.22)	-0.66 (-1.79, 0.48)
	Post-secondary or greater		1.54 (-0.29, 3.38)	-0.48 (-1.41, 0.46)	0.73 (-0.04, 1.5)	-1.37 (-3.32, 0.59)
Season	Early rainy	0.91	-0.14 (-1.01, 0.74)	0.17 (-0.69, 1.02)	0.55 (-0.17, 1.26)	-0.19 (-1.35, 0.97)
	Late rainy		-0.44 (-1.19, 0.31)	-0.27 (-1.07, 0.52)	0.24 (-0.35, 0.83)	-0.97 (-2.58, 0.64)
	Early dry		-0.37 (-1.07, 0.34)	-0.31 (-1.07, 0.45)	0.15 (-0.44, 0.74)	-0.37 (-1.45, 0.71)
Underlying Determinants – Services						
Number of rooms in household		0.035	-0.1 (-0.39, 0.19)	-0.03 (-0.19, 0.14)	-0.19 (-0.41, 0.03)	0.31* (0.04, 0.58)
Household size		0.36	0.07 (-0.04, 0.18)	-0.01 (-0.12, 0.09)	0.04 (-0.07, 0.15)	0.17 (-0.02, 0.36)
Household has improved floor		0.98	0.18 (-0.67, 1.02)	0.14 (-0.38, 0.66)	0.25 (-0.32, 0.82)	0.33 (-0.65, 1.31)
Underlying Determinants – Practices						
Hygiene score		0.87	-0.06 (-0.27, 0.15)	-0.06 (-0.2, 0.07)	0 (-0.13, 0.13)	0.09 (-0.54, 0.72)
Food hygiene score		0.66	-0.67 (-3.14, 1.8)	0.08 (-0.22, 0.38)	-0.09 (-0.37, 0.2)	0.27 (-0.44, 0.98)
Animal exposure score		0.82	0.07 (-0.27, 0.4)	-0.02 (-0.24, 0.21)	-0.11 (-0.33, 0.11)	-0.12 (-0.56, 0.32)
Average maternal hand contamination†		0.32	1.04* (0.06, 2.02)	0.65 (-0.2, 1.51)	0.22 (-0.4, 0.84)	-0.43 (-1.86, 1)
Underlying Determinants – Food						
Moderately/severely food insecure		0.95	-0.24 (-0.92, 0.44)	-0.37 (-0.92, 0.19)	-0.42 (-0.88, 0.04)	-0.12 (-1.27, 1.02)
Average drinking water contamination†		0.68	-0.07 (-1.82, 1.68)	-0.03 (-0.71, 0.66)	-0.34 (-0.88, 0.21)	0.49 (-0.83, 1.81)

Note: *** p.value < 0.001; ** p.value < 0.01; * p.value < 0.05; aMD = adjusted mean difference; CI = confidence interval; LGAZ = length-for-gestational-age Z-score; ΔLAZ = change in length-for-age Z-score. Models are adjusted using the UNICEF categories: Model 1 includes Governance only, Model 2 includes all *Enabling Determinants*, Model 3 = all *Enabling Determinants* and *Underlying Determinants* except (†) variables, Model 4 = all *Enabling Determinants* and *Underlying Determinants, Model 5* = all *Enabling, Underlying,* and *Immediate Determinants.* All models are adjusted for year of measurement, and 3-month ΔLAZ models are adjusted for days between measurement, age at the beginning of the interval, and LAZ at the beginning of the interval.

Table S5. Effect modification of the association between community, demographic, and environmental exposures and postnatal growth trajectories by child age.

Variable	Contrast	Global p-value	Stratified Estimates 0-6 months	Stratified Estimates 7-24 months
Enabling Determinants – Governance				
Community type	Intermediate	0.00027	-0.16 (-0.32, 0.01)	-0.13 (-0.27, 0.01)
	Rural - road		-0.05 (-0.2, 0.11)	-0.16* (-0.3, -0.03)
	Rural - river		-0.17 (-0.39, 0.06)	-0.64*** (-0.86, -0.42)
Enabling Determinants – Norms				
Child is male		0.74	-0.11 (-0.23, 0)	-0.13** (-0.23, -0.03)
Birth order	2	0.84	0.05 (-0.12, 0.22)	0.02 (-0.11, 0.16)
	3+		-0.03 (-0.22, 0.16)	-0.07 (-0.23, 0.08)
Maternal age during pregnancy	22-29 years	0.042	-0.11 (-0.25, 0.03)	-0.09 (-0.23, 0.04)
	Less than 22 yrs		-0.12 (-0.31, 0.07)	0.07 (-0.12, 0.25)
Mom identifies as Afro-Ecuadorian		0.0033	0.15 (0, 0.3)	-0.02 (-0.16, 0.11)
Enabling Determinants – Resources				
Household wealth quartile	Q2	0.54	0.15 (-0.02, 0.33)	0.09 (-0.05, 0.23)
	Q3		0.08 (-0.08, 0.24)	0.14 (-0.02, 0.3)
	Q4		0.18* (0.02, 0.34)	0.14* (0.01, 0.27)
Maternal height (per 5 cm)		0.59	0.09*** (0.04, 0.14)	0.1*** (0.06, 0.14)
Maternal education	Lower secondary	0.43	-0.07 (-0.32, 0.19)	-0.13 (-0.35, 0.09)
	Upper secondary		-0.13 (-0.35, 0.09)	-0.05 (-0.25, 0.15)
	Post-secondary or greater		-0.24 (-0.48, 0)	-0.15 (-0.36, 0.06)
Season	Early rainy	0.25	0 (-0.16, 0.16)	0.13* (0.01, 0.25)
	Late rainy		0.07 (-0.1, 0.24)	0.08 (-0.01, 0.18)
	Early dry		-0.03 (-0.18, 0.11)	0.1* (0.01, 0.2)
Underlying Determinants – Services				
Number of rooms in household		0.33	0 (-0.05, 0.05)	-0.03 (-0.07, 0.02)
Household size		0.58	0 (-0.02, 0.03)	0.01 (-0.02, 0.03)
Household has improved floor		0.72	-0.02 (-0.16, 0.12)	0 (-0.12, 0.13)
Household has improved toilet		0.29	-0.19 (-0.49, 0.11)	-0.29* (-0.57, -0.01)
Underlying Determinants – Practices				
Hygiene score		0.099	0.01 (-0.03, 0.04)	-0.02 (-0.05, 0.01)
Food hygiene score		0.67	0.02 (-0.06, 0.11)	0.04 (-0.04, 0.12)
Animal exposure score		0.94	-0.05 (-0.11, 0.01)	-0.05* (-0.09, -0.01)
Average maternal hand contamination†		0.0043	-0.09 (-0.28, 0.1)	0.14 (-0.02, 0.3)
Underlying Determinants – Food				
Moderately/severely food insecure		0.58	0.05 (-0.06, 0.16)	0.01 (-0.07, 0.1)
Child was exclusively breastfed for >50% of days 0-6m		0.8	0.1 (-0.02, 0.23)	0.09 (-0.02, 0.19)
Average drinking water contamination†		0.098	-0.04 (-0.19, 0.11)	0.08 (-0.08, 0.23)
Immediate Determinants – Care				
Average child hand contamination		0.0029	-0.18* (-0.35, -0.02)	0.02 (-0.13, 0.17)

Note: *** p.value < 0.001; ** p.value < 0.01; * p.value < 0.05; aMD = adjusted mean difference; CI = confidence interval; LGAZ = length-for-gestational-age Z-score; ΔLAZ = change in length-for-age Z-score. Models are adjusted using the UNICEF categories: Model 1 includes Governance only, Model 2 includes all *Enabling Determinants*, Model 3 = all *Enabling Determinants* and *Underlying Determinants* except (†) variables, Model 4 = all *Enabling Determinants* and *Underlying Determinants, Model 5* = all *Enabling, Underlying,* and *Immediate Determinants.* All models are adjusted for year of measurement, and 3-month ΔLAZ models are adjusted for days between measurement, age at the beginning of the interval, and LAZ at the beginning of the interval.

Table S6. Effect modification of the association between community, demographic, and environmental exposures and postnatal growth trajectories by community type.

			Stratified Estimates			
Variable	Contrast	Global p-value	Urban	Intermediate	Rural - road	Rural - river
Enabling Determinants – Norms						
Child is male		0.076	0.01 (-0.2, 0.21)	-0.22** (-0.38, -0.06)	-0.19** (-0.33, -0.06)	0.14 (-0.15, 0.43)
Birth order	2	0.38	-0.07 (-0.32, 0.18)	0.13 (-0.06, 0.32)	-0.05 (-0.27, 0.16)	0.15 (-0.39, 0.69)
	3+		-0.32* (-0.62, -0.02)	0.08 (-0.16, 0.32)	-0.07 (-0.27, 0.13)	0.05 (-0.39, 0.5)
Maternal age during pregnancy	22-29 years	0.75	0.01 (-0.24, 0.26)	-0.24* (-0.48, 0)	-0.04 (-0.21, 0.13)	-0.2 (-0.53, 0.13)
	Less than 22 yrs		0.11 (-0.17, 0.4)	-0.13 (-0.41, 0.14)	0.05 (-0.19, 0.29)	-0.11 (-0.67, 0.46)
Mom identifies as Afro-Ecuadorian		3.1e-35	0.01 (-0.2, 0.22)	-0.03 (-0.19, 0.14)	0.37** (0.14, 0.59)	-1.3*** (-1.49, -1.11)
Enabling Determinants – Resources						
Household wealth quartile	Q2	0.72	-0.04 (-0.32, 0.25)	0.28* (0.06, 0.51)	0.05 (-0.16, 0.26)	0.07 (-0.38, 0.52)
	Q3		0.08 (-0.17, 0.33)	0.23 (-0.06, 0.52)	0.02 (-0.17, 0.21)	0.05 (-0.45, 0.54)
	Q4		0.15 (-0.09, 0.39)	0.29** (0.09, 0.49)	0.02 (-0.18, 0.22)	-0.08 (-0.67, 0.51)
Maternal height (per 5 cm)		0.86	0.11* (0.02, 0.21)	0.07* (0.01, 0.14)	0.09*** (0.04, 0.14)	0.12* (0, 0.24)
Maternal education	Lower secondary	0.015	0.58 (-0.06, 1.23)	-0.11 (-0.5, 0.27)	-0.03 (-0.31, 0.25)	-0.4* (-0.76, -0.05)
	Upper secondary		0.62* (0.07, 1.17)	0.13 (-0.18, 0.44)	-0.06 (-0.33, 0.2)	-0.69*** (-1.09, -0.28)
	Post-secondary or greater		0.57 (-0.05, 1.19)	0.02 (-0.31, 0.34)	-0.24 (-0.53, 0.04)	-0.45* (-0.84, -0.07)
Season	Early rainy	0.00024	0.04 (-0.13, 0.22)	0.01 (-0.15, 0.17)	0.24** (0.08, 0.4)	-0.19 (-0.43, 0.04)
	Late rainy		0.05 (-0.12, 0.23)	-0.08 (-0.23, 0.07)	0.32*** (0.19, 0.45)	-0.27* (-0.53, -0.01)
	Early dry		-0.03 (-0.2, 0.14)	-0.07 (-0.21, 0.07)	0.22** (0.09, 0.35)	-0.08 (-0.28, 0.12)
Underlying Determinants – Services						
Number of rooms in household		0.4	-0.07 (-0.15, 0.02)	0.01 (-0.05, 0.06)	-0.01 (-0.09, 0.06)	-0.04 (-0.12, 0.03)
Household size		0.91	0 (-0.03, 0.04)	0 (-0.03, 0.03)	0 (-0.03, 0.04)	0.02 (-0.03, 0.08)
Household has improved floor		0.98	-0.03 (-0.22, 0.16)	0 (-0.18, 0.18)	0.01 (-0.19, 0.21)	-0.06 (-0.4, 0.28)
Underlying Determinants – Practices						
Hygiene score		0.027	-0.04 (-0.11, 0.03)	0.01 (-0.04, 0.05)	0 (-0.04, 0.04)	-0.27** (-0.45, -0.09)
Food hygiene score		8.5e-15	0.99*** (0.77, 1.22)	0.02 (-0.06, 0.11)	0 (-0.1, 0.1)	0.19* (0.02, 0.35)
Animal exposure score		0.81	-0.1 (-0.2, 0.01)	-0.05 (-0.11, 0.01)	-0.05 (-0.1, 0)	-0.01 (-0.18, 0.15)
Average maternal hand contamination†		0.42	-0.01 (-0.39, 0.37)	0.25 (-0.08, 0.58)	0.01 (-0.19, 0.21)	-0.2 (-0.63, 0.24)
Underlying Determinants – Food						
Moderately/severely food insecure		0.0043	0 (-0.13, 0.14)	-0.01 (-0.14, 0.12)	0.01 (-0.11, 0.13)	0.4*** (0.2, 0.6)
Child was exclusively breastfed for >50% of days 0-6m		0.12	-0.07 (-0.29, 0.15)	0.03 (-0.14, 0.2)	0.2** (0.06, 0.35)	0.3 (-0.07, 0.68)
Average drinking water contamination†		0.23	0.49 (-0.26, 1.25)	0.13 (-0.14, 0.4)	-0.01 (-0.17, 0.15)	-0.34 (-0.82, 0.14)
Immediate Determinants – Care						
Average child hand contamination		0.52	-0.25 (-0.6, 0.1)	-0.14 (-0.48, 0.21)	-0.06 (-0.21, 0.09)	0.19 (-0.27, 0.65)

Note: *** p.value < 0.001; ** p.value < 0.01; * p.value < 0.05; aMD = adjusted mean difference; CI = confidence interval; LGAZ = length-for-gestational-age Z-score; ΔLAZ = change in length-for-age Z-score. . Models are adjusted using the UNICEF categories: Model 1 includes Governance only, Model 2 includes all *Enabling Determinants*, Model 3 = all *Enabling Determinants* and *Underlying Determinants* except (†) variables, Model 4 = all *Enabling Determinants* and *Underlying Determinants, Model 5* = all *Enabling, Underlying,* and *Immediate Determinants.* All models are adjusted for year of measurement, and 3-month ΔLAZ models are adjusted for days between measurement, age at the beginning of the interval, and LAZ at the beginning of the interval.
